# Primates as Predictors of Mammal Community Diversity in the Forest Ecosystems of Madagascar

**DOI:** 10.1371/journal.pone.0136787

**Published:** 2015-09-03

**Authors:** Kathleen M. Muldoon, Steven M. Goodman

**Affiliations:** 1 Department of Anatomy, Arizona College of Osteopathic Medicine, Midwestern University, Glendale, Arizona, United States of America; 2 Field Museum of Natural History, 1400 South Lake Shore Drive, Chicago, Illinois, United States of America; 3 Association Vahatra, BP 3972, Antananarivo, Madagascar; Texas A&M University, UNITED STATES

## Abstract

The geographic distribution of species is the typical metric for identifying priority areas for conservation. Since most biodiversity remains poorly studied, a subset of charismatic species, such as primates, often stand as surrogates for total biodiversity. A central question is therefore, how effectively do primates predict the pooled species richness of other mammalian taxa? We used lemurs as indicator species to predict total non-primate mammal community richness in the forest ecosystems of Madagascar. We combine environmental and species occurrence data to ascertain the extent to which primate diversity can predict (1) non-primate mammal α-diversity (species richness), (2) non-primate complementarity, and (3) non-primate β-diversity (species turnover). Our results indicate that primates are effective predictors of non-primate mammal community diversity in the forest ecosystems of Madagascar after controlling for habitat. When individual orders of mammals are considered, lemurs effectively predict the species richness of carnivorans and rodents (but not afrosoricids), complementarity of rodents (but not carnivorans or afrosoricids), and all individual components of β-diversity. We conclude that lemurs effectively predict total non-primate community richness. However, surrogate species alone cannot achieve complete representation of biodiversity.

## Introduction

Understanding the relationship between patterns of community structure and the measurement and monitoring of biodiversity is a central goal of conservation biogeography [1]. Information on species diversity and distribution is widely used for setting conservation priorities [2–4], prioritizing new reserve sites [5, 6], and conservation management [7]. However, most biodiversity remains undescribed (a knowledge gap known as the “Linnaean shortfall”), and the geographic distribution of most species is poorly understood (the “Wallacean shortfall”) [8]. Thus, the use of incomplete information to indicate how and where conservation efforts should be concentrated is a major obstacle to protecting tropical habitats [9]. One widely adopted solution to this problem is to select priority areas based on protection afforded to one or more taxonomic groups, with the assumption that those reserves will also conserve a broader array of organisms (e.g., see review in [10]). The validity of this assumption depends on how well the chosen surrogate group represents overall biodiversity, as well as levels of species turnover and habitat heterogeneity in the region of interest. Thus, selecting surrogates is an integral part of successful conservation planning [11].

Surrogate species are usually taxa whose diversity and distributions are already well known, or are relatively easily determined. Numerous studies have evaluated the performance of potential surrogates spanning a broad taxonomic spectrum (see review in [12]). Across these studies, indicator groups are generally considered effective surrogates for lesser-known biodiversity if there is cross-taxon congruence in species richness hotspots (i.e., α-diversity [13–16]), or patterns of species turnover (i.e., β-diversity or complementarity [17–19]). The difference between these methods is that rather than just selecting the set of sites with the highest species richness, which can involve redundant representation, the goal of a complementarity-based approach is to select sites that show the greatest biotic differences in their taxonomic composition. In other words, a complementarity approach aims to select sites that differ greatly in represented species, and therefore protect more taxa in combination. Complementarity often encompasses organisms that have micro-endemic distributions.

Results from previous studies have been mixed, casting doubt on whether general patterns of cross-taxon congruence in spatial distributions exist. At broad spatial scales, most studies indicate there is concordance in the distribution of species richness between taxa, e.g. globally [20], in biodiversity hotspots [4], in WWF’s ecoregions [3, 21], in the tropics [22], and in sub-Saharan Africa [15]. At fine spatial scales, cross-taxon congruence patterns between groups at the family level are more ambiguous; sometimes showing low [9, 13] and in other cases high congruence [17], depending on the ecological or taxonomic similarity or of included taxa [23, 24]. This discrepancy has led to systematic investigations of the factors that influence the performance of surrogate groups (e.g., [12, 18]). It may be impossible, or at least impractical, to make universal generalizations because evolutionary history and the impact of human pressure differ so drastically from region to region.

In many areas of the world, particularly the tropics, information on primate distribution is often more commonly available than for other taxa because primates are relatively easily surveyed [25] and taxonomically well-known [26]. In addition, owing to the broad public appeal of primates, general support for their conservation is often greater than for other taxa. Consequently, as a single-taxonomic group surrogate, primates drive conservation efforts in many tropical areas [27]. It is therefore striking that few studies have analyzed the effectiveness of primates as a biodiversity surrogate (but see [25, 28, 29]).

In this study, we focus on the use of lemurs, the endemic primates of Madagascar, as surrogates of non-primate mammal biodiversity in forested habitats. Madagascar is one of the highest conservation priorities in the world, due to its high number of endemic plants and animals, and the increasing human pressure on its natural ecosystems [30]. Lemurs are widely recognized to be the flagship taxa for biodiversity conservation in Madagascar [31]. Indeed, until a few decades ago, faunal exploration on the island focused almost exclusively on lemurs [32]. Lemurs are potentially good surrogates for non-primate mammal biodiversity because they are ecologically diverse, forest-dependent, and they occur in all natural terrestrial habitats of Madagascar—often in relatively high species densities [29, 33]. In some areas, species densities were higher in the recent past [34], although this phenomenon is not island-wide, and is mediated by forest fragmentation, vegetation type, and human impact (e.g., [35]). Lemurs are the most easily inventoried of Madagascar’s mammals, particularly the diurnal taxa, which are relatively large, conspicuous, and noisy. Visual transect surveys of lemurs usually reach species accumulation curve asymptotes within 30 hours or less of fieldwork at a site (e.g., [36–38]). In contrast, during small mammal inventories in the same forests, when rodents or afrosoricids are captured, an asymptote is not usually reached for four or five days of intensive sampling (e.g., [39–44]). It is therefore of practical importance to conservation efforts on Madagascar to know if lemurs are useful predictors of the other segments of the mammalian assemblage.

General patterns of cross-taxon congruence in species richness or endemism have not been established on Madagascar. Previous studies have shown conflicting results, finding, for example, that lemurs perform poorly as biodiversity surrogates for non-primate mammals [29, 45], or well for only certain portions of the fauna [25]. These results may have been due to the use of partial data sets to test prediction hypotheses. A large amount of information is now available on the species limits, biology, and distribution of Malagasy mammals. These data allows us to revisit the use of lemurs as predictors of non-primate mammals in the forest ecosystems of Madagascar [31, 46]. Recent work, based on a considerable database, has also demonstrated that the biogeographic distributions of extant mammal assemblages on Madagascar are structured according to current habitat characteristics [47, 48]. These findings suggest that extant mammalian distribution patterns on Madagascar maintain a signal that at least partly reflects the original geography of speciation and, by consequence, that they are robust units for biogeographic analyses on Madagascar.

Our objective is to evaluate the extent to which primate diversity predicts non-primate mammal diversity in the forested ecosystems of Madagascar. We ask, is primate diversity a significant predictor of non-primate mammal diversity after controlling for habitat? We combine environmental and species occurrence data to ascertain the extent to which primate diversity can predict (1) non-primate mammal α-diversity (species richness), (2) non-primate complementarity, and (3) non-primate β-diversity (species turnover). The results of our study have implications for the parameters used in protected area selection on Madagascar.

## Materials and Methods

### Datasets

We used species lists of endemic terrestrial mammals from 30 forested areas [21] excluding bats and, domestic and exotic species (e.g., Soricidae shrews and Muridae rodents). The number of lemur species recognized on Madagascar has increased considerably over the last two decades, with summary tabulations of 32 species in 1994 [49], 71 in 2006 [50], and 97 in 2010 [31]. A considerable portion of these new taxa have been named exclusively or largely on molecular genetic data. In many cases, there are taxonomic complications with these descriptions, such as lack of sequence data from topotypic material, problems associated with sample sizes and geographic coverage, and differentiating between clinal genetic variation and distinct phylogenetic species between allopatric populations (e.g., [51]). While it was not our intent to evaluate the validity of these recently named lemurs, in order to analyze correctly the predictive relationships between lemurs and other mammal taxa, we were obliged to consider the species epithet associated with the presence of a taxon at a given site. We accomplished this in two manners: 1) by accepting the lemur taxonomy presented in Mittermeier et al. [31], regardless of whether some species described on the basis of genetic characteristics are valid; and 2) by removing recently named taxa that did not respect one of the following criteria: A) both mitochondrial and nuclear genes were used in the analysis, with the geographically closest lying samples defining a clade not separated by more than 200 km; or B) if the above rule was not met and the results were largely or exclusively based on mtDNA, at least 10 samples per new taxa had to be included, which is less conservative than the 10 individuals per locality proposed by Markolf et al. [51].

Our study included 141 mammal species within the orders Primates (PRI), Carnivora of the Eupleridae (CAR), Afrosoricida of the Tenrecidae (AFR), and Rodentia of the Nesomyinae (ROD). For each forested area, we calculated total non-primate mammal (TLNP) community richness as the sum of all species occurrences within the above orders, excluding PRI, which was the predictor group (see Table A in S1 File).

**Fig 1:**
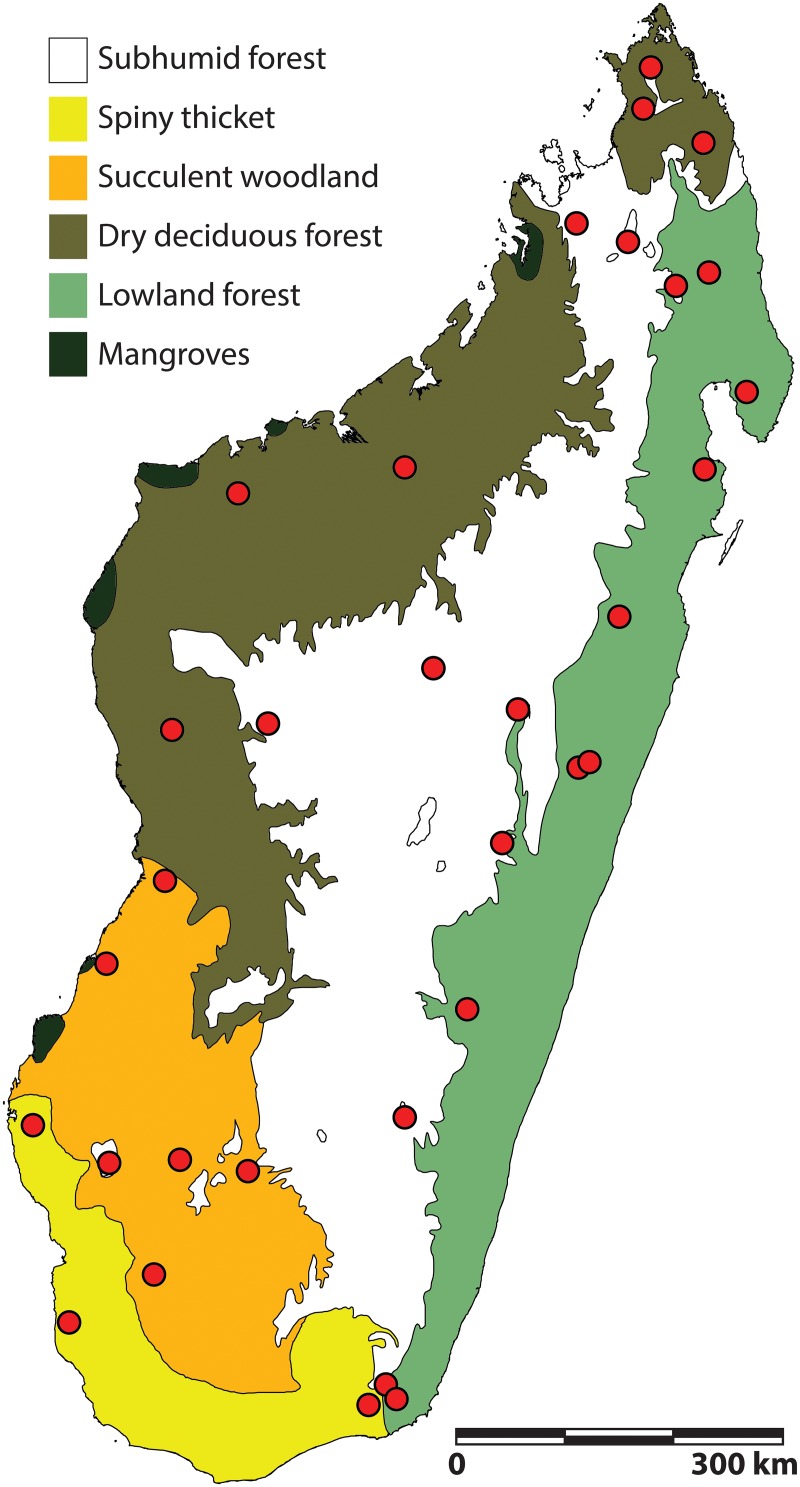
Map of terrestrial ecoregions of Madagascar. Circles indicate geographic locations of the 30 localities used in the community diversity analyses.

To account for spatial autocorrelation in our analysis and as a proxy for environmental variables, we used the World Wildlife Fund's classification of Madagascar’s natural habitats (2). This system divides Madagascar into seven ecoregions that are broadly associated to amount of rainfall, length of dry season, and plant community structure: spiny thicket, succulent woodland, dry deciduous forest, subhumid forest, humid forest, ericoid thicket, and mangroves (Fig 1, Table B in S1 File). We excluded ericoid thicket and mangroves from our analysis because the mammal groups analyzed in our dataset and occurring in these habitats have not been uniformly surveyed or reported in the literature. We use ecoregions rather than continuous environmental data for several reasons. Globally, ecoregions serve as the basis for biodiversity hotspot identification and conservation planning (e.g., [4, 52, 53]). Moreover, the climatic and habitat variables captured by ecoregions are important components to understand the biogeography and evolutionary history of extant Malagasy mammals (e.g., [21, 54]). However, substantial microhabitat variation exists within each ecoregion, and there has been massive anthropogenic degradation of the existing native habitats on Madagascar [55, 56]. A specific habitat type dominates most forest communities included in our analysis, but virtually none exclusively represents a single habitat. Although the use of ecoregions as a proxy for environmental variables masks microhabitat heterogeneity, these categories represent habitats that correspond to patterns of ecological organization [21]. Therefore, we consider the ecoregion model applicable to broad-scale comparisons between mammal communities.

In most cases, forests selected for our analysis were inventoried by the same researchers, ensuring similar techniques and levels of effort (e.g., [57–62]). The data available for the non-primate mammal fauna of surveyed sites limited our sample size. Following Muldoon and Goodman [21], we considered forested zones that span a significant elevational gradient to contain non-continuous mammal communities, and separated the communities as the humid (0–800 m) and subhumid (800–1600 m) zones (2). Our comparative sample included 34 mammal communities from 30 localities (Fig 1).

### Analysis

Prior to evaluating primates as predictors of non-primate community richness, we tested the effect of area on TLNP species richness of each community using linear least squares regression analysis. Although the selected localities vary widely in area, ranging from 265 to 230,000 ha of protected land, we found no significant correlation between area and any measure of richness. This is consistent with previous results indicating that at a continental scale, environment and history are more important than area in determining species richness [21, 63, 64], and that the smallest blocks used in this study maintain intact species communities, at least in the short-term. When comparing species richness, we therefore used raw species counts instead of residuals from the area regression.

To evaluate primate species richness as a predictor of mammal community richness on Madagascar, we used multiple linear regression analysis [65]. The purpose of regression analysis is to describe the dependence of a variable Y (in this case, TLNP, for example) on an independent X (e.g., primates). In other words, regression is most appropriate for purposes of prediction, of Y in terms of X, and for purposes of explaining some of the variation of Y by X, by using the latter variable as a statistical control [65]. The use of correlation analysis is most appropriate when the hypothesis is whether two variables are interdependent, or co-vary (e.g., there is no distinction between independent and dependent variables). Although similar studies have employed correlation analysis [3, 66, 67], regression is a more appropriate statistical tool for purposes of prediction [23, 68–70]. Two sets of predictor variables were entered hierarchically into the model: ecoregion was dummy-coded and entered into the analysis first (Block 1) based on previous results demonstrating a relationship between ecological organization and habitat type on Madagascar [21]; primate species richness was then entered into the analysis (Block 2). We examined the regression coefficient (R^2^), as well as the residuals from the regression by calculating two additional parameters: Cook’s distance (*D*_*i*_), a regression diagnostic that provides a comparative index of the leverage of a particular community on the analysis; and the mean percentage prediction error (% PE). We removed outliers from the analysis if *D*_*i*_ > 4 / *n*, where *n* = 34 (i.e., the number of mammal communities, [65, 71]). Individual prediction errors were calculated as ([observed-predicted]/predicted) x 100) [70, 72]. We used PRI as indicator species to predict richness within our four reference groups: (1) TLNP; (2) CAR; (3) AFR; and (4) ROD. We adjusted p-values for multiple comparisons using Bonferroni’s correction. Statistical analyses were performed using SPSS 22.0 [73].

Complementarity exists when an area has biodiversity features (e.g., species) that are unrepresented in another area with which it is being compared [74]. For example, a patch of humid forest and a patch of desert might each be expected to have a higher number of different species (and so will represent more species in combination) than would two different patches within the same kind of humid forest [75]. In other words, complementarity increases when one community has microendemic species that are not present in other communities. We calculated two measures of complementarity. First, we quantified complementarity *sensu* Williams et al. [75] as the species richness of a focal biota (e.g., TLNP, PRI, CAR, AFR, and ROD) for one community (e.g., community A) that was unrepresented in the species richness of every other community in the analysis (e.g., this measure was calculated for comparisons with community B, community C, etc.) (Tables C-G in S1 File). We then plotted this complementarity measure for the surrogate (e.g., PRI) on the x-axis, against the corresponding complementarity for predicted component of the fauna (e.g., TLNP, CAR, AFR, or ROD) of the same community on the y-axis. A strength of this approach is that it acknowledges that complementarity between faunal pairs is usually asymmetrical [75, 76]. For example, Ganzhorn [21] documented that species-poor lemur communities represent nested subsets of species-rich communities in both eastern humid and western dry forests on Madagascar, regardless of habitat characteristics or forest area. In this case, the species-rich community would have many species that are unrepresented in the species-poor community (high complementarity), but the species-poor community would have no species that are not represented in the species-rich community (low complementarity). Because of this asymmetry, we could examine comparisons in either direction between pairs of communities: i.e., the complementarity *of* each community in turn to all of the other cells, or the complementarity *to* each community in turn of all the other communities. We follow Williams et al. [73], and view the complementarity *of* each community as being more relevant to building conservation networks (Tables C-G in S1 File). We calculated a mean complementarity *of* value for each community, and used multiple linear regression analysis as described above to test the hypothesis that complementarity within primates can predict complementarity in non-primate mammals on Madagascar.

Using complementarity *sensu* Williams et al. [75], for a set of n communities, there are n^2^ –n comparisons. When dealing with even moderate numbers of communities, the number of comparisons becomes very large (e.g., for our 34 communities, there were more than 1000 comparisons for each of the two groups). In a practical context, this is a time-consuming set of computations [76]. Furthermore, standard statistical tests of significance cannot be applied to full complementarity measures because the matrix is asymmetrical. Therefore, we also calculated β-diversity (species turnover among communities) by using the Marczewski-Steinhaus (MS) distance, which is the complement of the standard Jaccard similarity index [77]. Although complementarity was not explicitly introduced as a concept of β-diversity, recent work on ecological diversity recognizes it as such: the more complementary two sites are, the greater the rate of species turnover between sites, and the higher their β-diversity [9, 15, 17, 76–78]. Because MS distance is a metric measure, it can be treated as a distance index and used in regression analysis [77]. Therefore, to test the hypothesis that β-diversity of primates predicts β-diversity of non-primate mammals after controlling for habitat, we conducted multiple matrix regression analysis (with 10000 random permutations). Multiple matrix regression analyses were conducted using the program multi_mantel [79], which performs hypothesis testing of the multiple regression by permuting rows and columns together in the dependent matrix, following the Mantel permutation procedure [80].

## Results

### General Sample Characteristics

The subhumid forest ecoregion had the highest total mammal species richness, followed by humid forest, dry deciduous forest, succulent woodland, and spiny thicket (Table 1). PRI were the most speciose order per mammalian community in all ecoregions, with the exception of subhumid forest, where AFR had the highest species richness. Species richness was significantly higher for AFR in the mid-elevation forests of the subhumid forest ecoregion than in the drier ecoregions of the west. CAR were the least speciose order per mammal community in the drier ecoregions of the west, whereas ROD were the least speciose order per community in subhumid and humid forests.

**Table 1:** Species richness (r) and complementarity (c) per mammal community^a^. Mean values are given for each ecoregion.

Locality	rPRI	rCAR	rAFR	rROD	rTLNP	cPRI	cCAR	cAFR	cROD	cTLNP
Tsimanampetsotsa	4	2	4	2	8	5	1	2	1	5
Beza Mahafaly	4	1	4	2	7	4	0	2	2	4
Berenty	6	1	4	1	6	6	0	2	1	3
Andohahela Parcel 2	7	1	3	1	5	6	0	2	1	2
Mikea	8	2	5	2	9	7	1	3	1	6
***mean (spiny thicket)***	**5.8**	**1.4**	**4.0**	**1.6**	**7.0**	**5.6**	**0.4**	**2.2**	**1.2**	**4.0**
Kirindy CFPF	8	2	7	3	13	7	1	4	3	8
Zombitse-Vohibasia	8	1	5	2	8	8	0	3	2	5
Kirindy-Mitea	8	2	5	2	9	5	1	2	1	5
Isalo	7	1	2	2	5	6	0	3	2	2
***mean (succulent woodland)***	**7.8**	**1.5**	**4.8**	**2.3**	**8.8**	**6.5**	**0.5**	**3.0**	**2.0**	**5.0**
Ankarana	11	4	3	2	9	10	2	1	2	5
Ankarafantsika	8	2	3	4	9	8	1	1	3	5
Namoroka	10	2	3	2	7	9	1	1	2	4
Bemaraha	11	2	3	4	11	10	1	1	3	5
Loky-Manambato	9	2	7	3	12	7	1	3	2	6
***mean (dry deciduous forest)***	**10.0**	**2.4**	**3.8**	**3.0**	**9.6**	**8.8**	**1.2**	**1.4**	**2.4**	**5.0**
Marojejy lowland	9	3	7	5	15	7	2	4	4	8
Masoala	10	6	10	4	20	9	4	7	3	11
Verezanantsoro	11	6	8	5	19	10	4	4	4	12
Zahamena	12	6	5	1	12	11	4	2	0	7
Mantadia	12	5	12	3	20	10	3	7	3	13
Andringitra lowland	10	4	6	5	15	8	2	4	4	9
Andohahela Parcel 1 lowland	8	4	8	5	17	7	2	5	4	10
***mean (humid forest)***	**10.3**	**4.9**	**8.0**	**4**	**16.9**	**8.9**	**3.0**	**4.9**	**3.1**	**10.0**
Montagne d’Ambre	7	4	9	3	16	6	3	5	2	8
Manongarivo subhumid	9	4	12	6	22	8	3	8	4	14
Tsaratanana subhumid	7	2	17	8	27	5	1	12	7	18
Marojejy subhumid	10	2	16	9	27	8	1	13	7	19
Anjanaharibe-Sud subhumid	11	3	16	11	30	9	1	12	10	22
Analamazaotra	12	4	11	7	22	10	1	8	6	14
Ambohitantely	4	1	11	1	13	3	0	7	0	8
Anjozorobe	11	5	17	8	30	9	0	12	7	19
Tsinjoarivo	11	4	16	5	25	0	2	12	4	16
Ranomafana	13	5	14	10	29	11	3	10	9	20
Andringitra subhumid	13	4	15	7	26	11	3	9	6	16
Andohahela Parcel 1 subhumid	6	5	14	6	25	5	3	10	0	12
Analavelona	7	1	4	1	6	6	0	3	2	3
***mean (subhumid forest)***	**9.5**	**3.5**	**13.5**	**6.6**	**23.6**	**7.7**	**1.5**	**9.3**	**4.9**	**24.2**

^a^PRI, primate; CAR, carnivoran; AFR, afrosoricid; ROD, rodent, TLNP, total non-primate.

### Species richness

**Fig 2:**
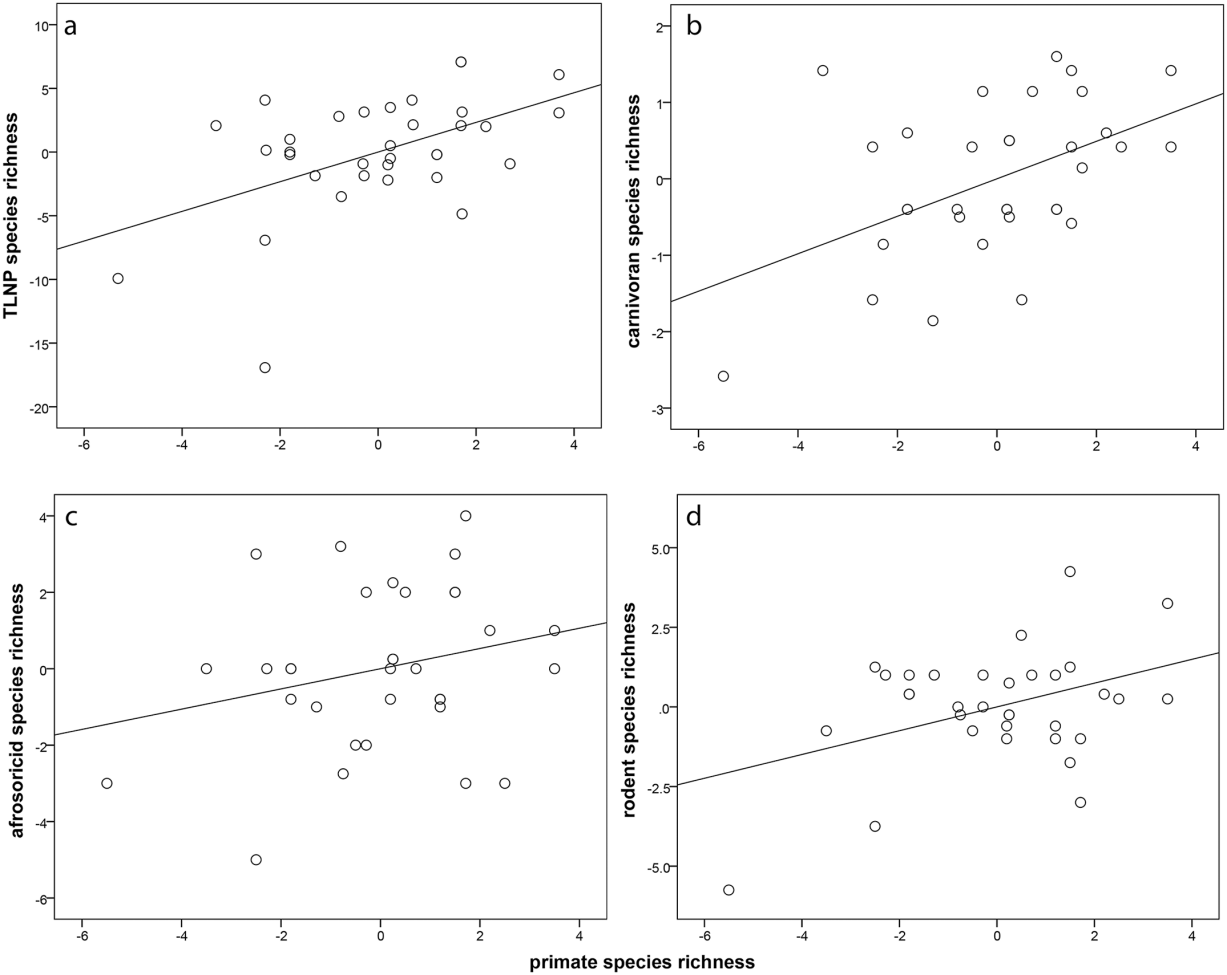
Partial regression plot of species richness across taxonomic groups on primate species richness. Line is least squares line of best fit. A. TLNP; B. CAR; C. AFR; D. ROD.

Initial examination of bivariate plots of primate species richness against total non-primate mammal community richness indicated an extreme outlier, Analavelona Forest. Although Analavelona is located in a semi-arid region, it supports a transitional humid-deciduous habitat, which Moat and Smith [81] recognized as a distinct forest type (western humid forest). This forest type is restricted to the upper 150 m of the 1320 m massif, with lower slopes supporting mostly anthropogenic grasslands, or vegetation more typical of the southwestern flora [81]. The mammalian community of the summital zone is typical of the dry southern portion of the island [21]. TLNP species richness of Analavelona was low, especially relative to other subhumid forests in our analysis (n = 6, Table 1). When comparing richness values of each mammalian order present at Analavelona, there was adequate representation of primates, despite underrepresentation of other groups, in particular carnivorans. One hypothesis is that during the last glacial maximum, climatic oscillations led to local small mammal extinctions during which forest species replaced endemic taxa. Furthermore, not much forest remains in the summital zone, which may have important impacts on carnivoran species richness. Leverage statistics indicated that Analavelona had undue influence on the results (*D*_*i*_ = 0.260, which exceeds the *a priori* exclusion criterion of *D*_*i*_ ≥ 0.125), with an absolute prediction error of 70.4%. Therefore, for the remaining analyses, we removed this community from our sample. The results of the analysis excluding Analavelona indicated that 85% (R^2^ = 0.850) of the variation in total non-primate mammal community richness was explained by ecoregion and primate species richness together. The majority of this variation was accounted for by ecoregion alone, while the unique contribution of primate species richness after controlling for ecoregion was 4.7% (F = 8.441, df = 1, 27, p < 0.01; Fig 2a, Table 2).

**Table 2:** Regression summary statistics for primates as predictors non-primate mammal species richness (r).

	Block	R^2^	SEE	R^2^ Δ	FΔ	df	Mean %PE
**rTLNP**	1	0.804	3.851	0.804	28.642***	4, 28	
	2	0.850	3.423	0.047	8.441**	1, 27	0.49
**rCAR**	1	0.608	1.088	0.608	10.878***	4, 28	
	2	0.698	0.973	0.090	8.504**	1, 27	0.24
**rAFR**	1	0.819	2.232	0.819	31.734***	4, 28	
	2	0.831	2.200	0.011	1.835	1, 27	0.24
**rROD**	1	0.554	1.966	0.554	8.691***	4, 28	
	2	0.627	1.831	0.073	5.275*	1, 27	3.48

* p< 0.05,

** p < 0.01;

*** p < 0.001; all values indicated by asterisks are also significant using Bonferroni’s correction.

Fig 2b and 2c illustrate the relationships between primate species richness and carnivoran and rodent species richness. In both cases, the predictive relationship was significant. Ecoregion alone accounted for the majority of this variation, while the unique contribution of primate species richness after controlling for ecoregion was 9.0% for carnivorans, and 7.3% for rodents (Table 2). The exception to the overall pattern was the regression of primate species richness on afrosoricid species richness (Fig 2d). The results of the analysis indicated that ecoregion explained 82% of the variation in afrosoricid richness, while primate species richness did not significantly contribute to the regression model (Table 2).

### Complementarity

**Fig 3:**
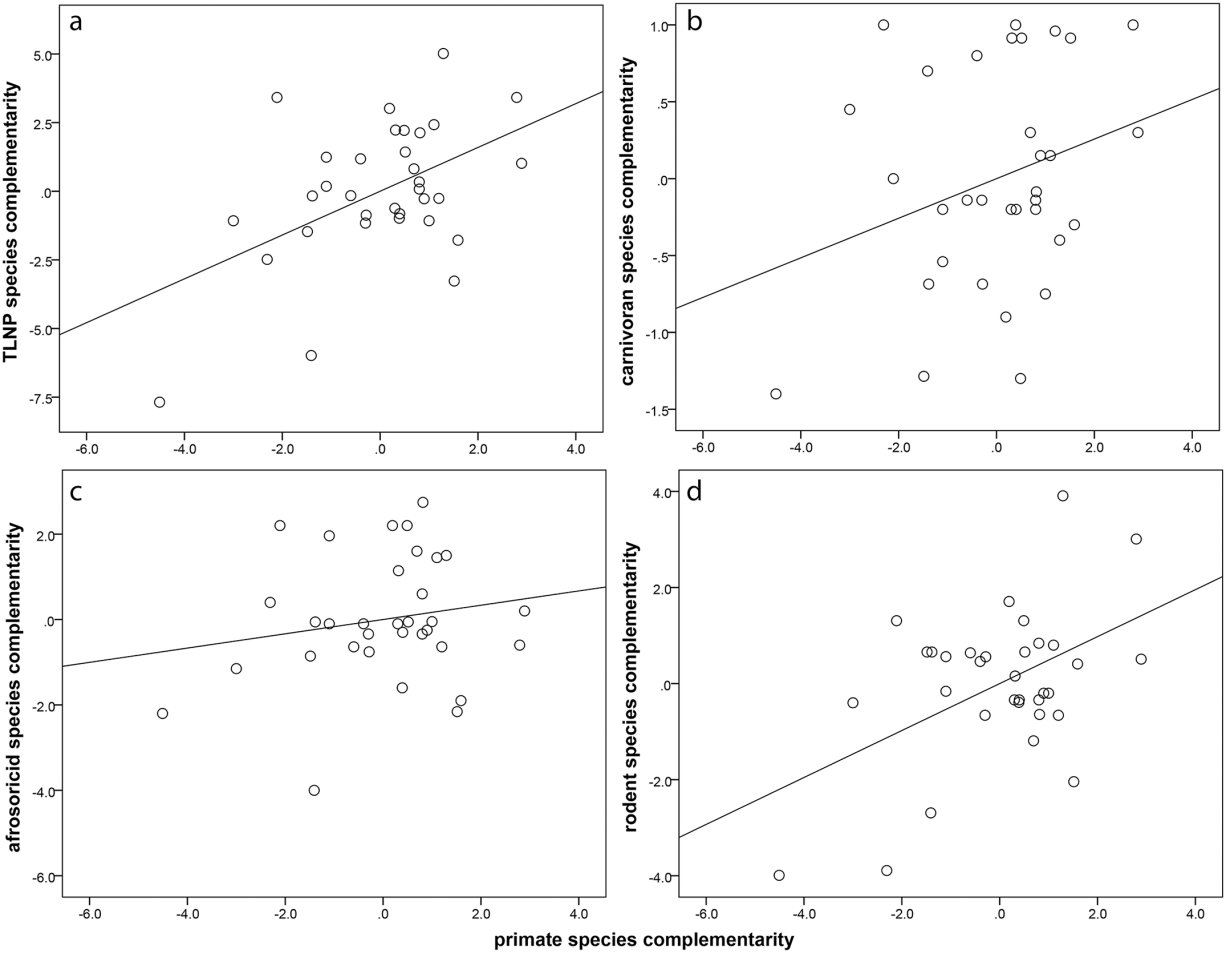
Partial regression plot of complementarity across taxonomic groups on primate species richness. Line is least squares line of best fit. A. TLNP; B. CAR; C. AFR; D. ROD.

Fig 3 illustrates the relationship between primate and non-primate complementarity. Our results indicated that 83% (R^2^ = 0.828) of the variation in total non-primate mammal complementarity was explained by ecoregion and primate complementarity together. The majority of this variation was accounted for by ecoregion alone, while the unique contribution of primate complementarity after controlling for ecoregion was 5.5% (F = 8.568, df = 1, 27, p < 0.01; Fig 3a, Table 3). When we analyzed the individual faunal components separately, the predictive relationship was significant for afrosoricids and rodents (Fig 3b and 3c). The majority of this variation was accounted for by ecoregion alone, while the unique contribution of primate complementarity after controlling for ecoregion was 0.6% for afrosoricids, and 13.2% for rodents (Table 3). Ecoregion alone explained 51.7% of the variation in carnivoran complementarity, while primate complementarity did not significantly contribute to the regression model (Fig 3d, Table 3).

**Table 3:** Regression summary statistics for primates as predictors non-primate mammal species complementarity (c).

	Block	R^2^	SEE	R^2^ Δ	FΔ	df	Mean %PE
**cTLNP**	1	0.773	2.728	0.773	23.888***	4, 28	
	2	0.828	2.421	0.055	8.568**	1, 27	1.93
**cCar**	1	0.517	0.765	0.517	7.565***	4, 28	
	2	0.556	0.747	0.039	2.345	1, 27	4.98
**cAFR**	1	0.807	1.587	0.807	29.288***	4, 28	
	2	0.813	1.591	0.006	0.872	1, 27	0.52
**cROD**	1	0.433	1.701	0.433	5.343**	4, 28	
	2	0.565	1.517	0.132	8.188**	1, 27	32.63

** p < 0.01;

*** p < 0.001;

All values indicated by asterisks are also significant with Bonferroni’s correction.

### Beta Diversity

**Fig 4:**
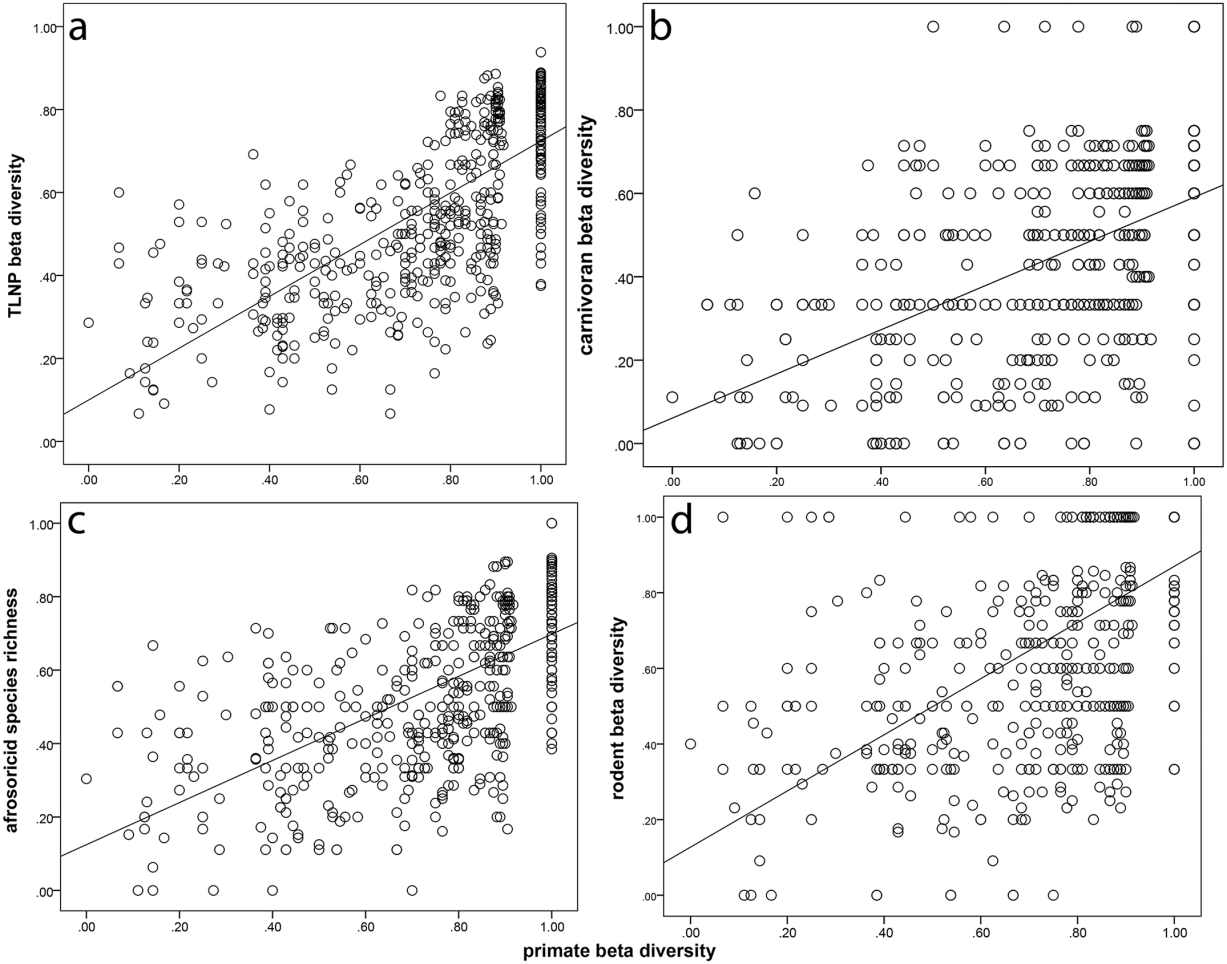
Partial regression plot of β-diversity across taxonomic groups on primate species richness. Line is least squares line of best fit. A. TLNP; B. CAR; C. AFR; D. ROD.

Fig 4 illustrates the relationship between the β-diversity of non-primate mammals and primates. Our results indicated that 49% (R^2^ = 0.512) of the variation in total non-primate mammal β-diversity was explained by ecoregion and primate β-diversity together (F = 14.768, df = 2, 525, p < 0.001; Fig 4a; Table 4). The majority of this variation was accounted for by primate β-diversity (R^2^ = 0.354). When individual faunal components were analyzed separately, the predictive relationship was significant in all cases (Fig 4b–4d, Table 4).

**Table 4:** Regression summary statistics for primates as predictors non-primate mammal β diversity (β).

	Block	R^2^	SEE	R^2^ Δ	FΔ	df	Mean %PE
**βTLNP**	1	0.158	0.017	0.158	98.737***	1, 526	
	2	0.490	0.031	0.332	342.487***	2, 525	0.40
**βCar**	1	0.008	0.026	0.008	4.224	1, 526	
	2	0.258	0.051	0.250	177.354**	2, 525	2.42
**βAFR**	1	0.227	0.018	0.227	154.546***	1, 526	
	2	0.451	0.035	0.224	215.322**	2, 525	0.28
**βROD**	1	0.082	0.023	0.082	47.104***	1, 526	
	2	0.319	0.045	0.237	183.332*	2, 525	1.49

* p< 0.05,

** p < 0.01;

*** p < 0.001;

All values indicated by asterisks are also significant with Bonferroni’s correction.

## Discussion

The goal of our study was to combine the parallel strengths of environmental, and species occurrence data to test the effectiveness of primates as surrogates for measures of non-primate diversity on Madagascar. On Madagascar, environmental data provide comprehensive spatial coverage of the island, and correlate with broad-scale patterns of mammalian community structure [21]. Conversely, biotic inventory data tend to be sparse in terms of spatial coverage across Madagascar, but provide the real biological entities necessary to measure biodiversity. When linked statistically, these data sources may be powerful predictors of spatial pattern in biodiversity [69, 82, 83].

Our results indicate that primates are effective predictors of non-primate mammal community diversity in the forest ecosystems of Madagascar after controlling for habitat. When individual orders of mammals are considered, lemurs effectively predict the species richness of carnivorans and rodents (but not afrosoricids), complementarity of rodents (but not carnivorans or afrosoricids), and all individual components of β-diversity. In contrast to primates, carnivorans generally have low complementarity values across Madagascar. Because they are secondary consumers, the species richness of their prey may not be as important as its abundance [84]. In other words, patterns of carnivoran diversity may not follow the same functional rules as primates. As a consequence, carnivoran complementarity may have little association with primate complementarity. An important exception to this point may be the fosa (*Cryptoprocta ferox*), the largest extant carnivoran on Madagascar. At some sites, more than 50% of their prey is lemur [85], indicating that there may be underlying patterns of co-distribution amongst certain predator-prey pairs. There is likely strong geographic bias in this relationship.

Afrosoricids demonstrate a mid-elevational peak in species richness in montane forest [59, 60, 86–91]. This pattern of diversity is reflected in the higher levels of total mammal richness in the subhumid forest when compared to the other ecoregions. Inspection of diversity values within individual faunal components substantiates this point, indicating that afrosoricids are the most species-rich order in the subhumid forest, in contrast to other ecoregions in which lemurs are the most speciose. Several different hypotheses have been proposed to explain the hump-shaped relationship between diversity and elevation, including environmental heterogeneity, competition, and historical factors [92–98]. The role of topographically complex areas on Madagascar as possible refugia and centers of speciation for small mammals during the Last Glacial Maximum deserves closer study [99].

Our results are in contrast to previous studies that have questioned the use of lemurs as surrogates for assessing biodiversity due to the effect of deterministic historical processes, such as selective extinction of vulnerable taxa during the Quaternary [100, 101]. Several points are important in this regard. There is ample evidence of human-caused transformation of the environment and associated fauna since human arrival on Madagascar more than 4000 years ago [56, 82, 102–104]. Following human arrival, the Quaternary extinction of numerous mammals largely resulted in the loss of similar functional components of mammal communities across all habitats for which fossil records are known (in general, large-bodied, diurnal, slow-reproducing, seed-dispersing primates) [105]. There are exceptions to the results of earlier studies—for example, recent discoveries and corrected attributions to *Hadropithecus* suggest it was functionally unique, and concentrated in the south and southwest [106, 107]. Furthermore, the notion of an exclusively megafaunal wave of extinction is probably an artifact of coarse historical paleontological techniques, which focused on the recovery of large-bodied lemurs. With new excavation techniques and finer details given to smaller bone, extinct small mammals are now being discovered [108]. Likewise, among still-extant mammals, habitat-specific range shifts and local extinctions were probably substantial in the Holocene [109–113]. It is likely that the factors that lead to either island-wide or local extinctions in the mammal fauna were consistent across the Quaternary communities, regardless of body-size. However, such paleocommunity analyses should be revisited in light of newly available genetic and biogeochemical evidence of extinct lemur diversity, and the paleodistributions of subfossil small mammals (e.g., [48, 113–115]).

### Applications of Results

Madagascar was one of the first tropical countries in the world to establish a reserve system [7]. The official legislation concerning natural resource conservation in the protected areas focuses on representative inclusion of all ecosystem types [7, 116]. In some cases, the discovery of specific taxon, such as the golden bamboo lemur, *Hapalemur aureus*, was the centerpiece for the creation of a national park [117]. This current study provides a further contribution in this context. We tested the effectiveness of primates as surrogates for non-primate mammal diversity across Madagascar in order to simplify the task of landscape-level biodiversity assessment and monitoring on Madagascar. Accurate conservation strategies require, when possible, information on the whole spectrum of taxonomic groups, at several scales, ranging from microhabitats to landscapes through regional to global. Although research on the small mammal faunas of Madagascar has recently improved [46], biodiversity surveys are rarely comprehensive enough to sample and identify all the species in a given area. Long-term ecological studies tend to focus on lemurs. The model applied here helps to overcome this limitation by combining species occurrence data of lemurs with environmental data to predict non-primate mammal diversity. We conclude that habitat type is a pragmatic basis for the assessment of mammal conservation priorities on Madagascar. In addition, lemurs effectively predict total non-primate community richness. However, surrogate species alone cannot achieve complete representation of biodiversity.

## Supporting Information

S1 File Raw data for analyses. Species richness data (Table A). Summary of geographic and climatic data used in the construction of the terrestrial ecoregions of Madagascar (Table B). Primate (PRI) complementarity data (Table C). Total non-primate (TLNP) complementarity data (Table D). Carnivoran (CAR) complementarity data (Table E). Afrosoricid (AFR) complementarity data (Table F). Rodent (ROD) complementarity data (Table G).
(XLS)
